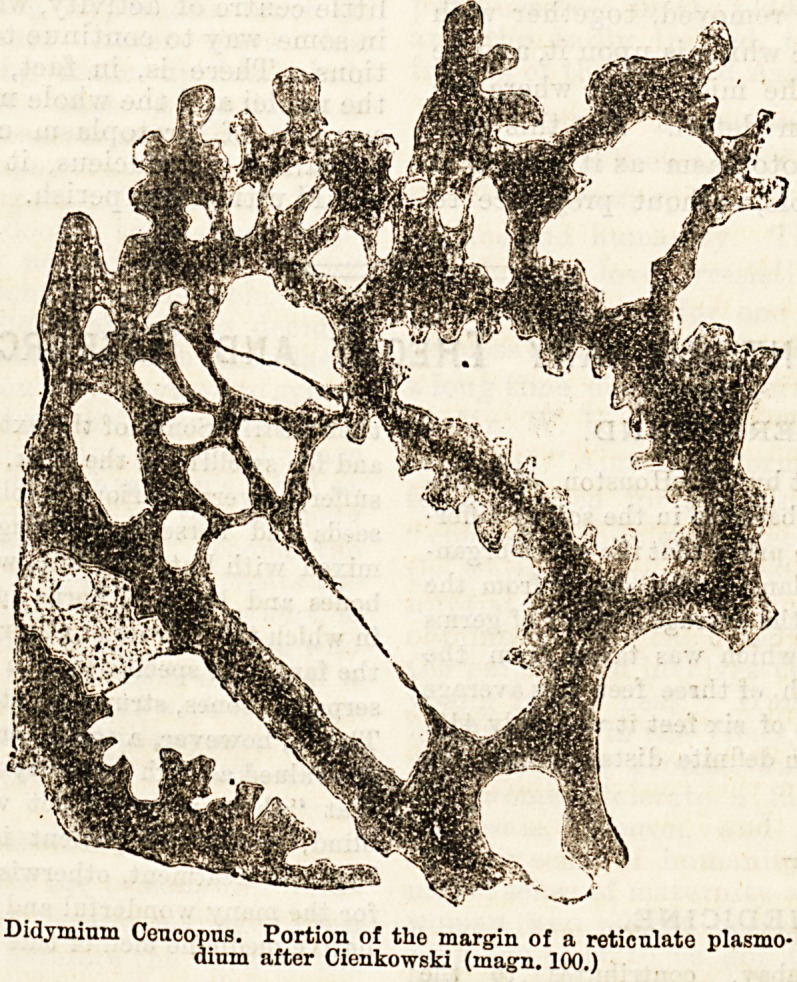# On the Cellular Structure of Living Organisms. I

**Published:** 1893-10-21

**Authors:** 


### On the Cellular Structure of Living Organisms.—I.

It is now a matter of common knowledge amongst most educated persons, that the bodies of animals and plants are made up of cells, but beyond this bare fact, perhaps the majority do not trouble to attempt to penetrate. One constantly hears vague statements about "Protoplasm," and so forth, from people who possess but hazy notions as to the real nature of the structure and substances whose names everybody knows.

The first impetus to the study of the cellular structure of organized life was imparted by the investigation of Malpighi, an Italian, and Grew, an Englishman, who, more than two hundred years ago, were led to recognize a chambered structure in the plant tissues which they examined. It is worthy of remark that in this instance, as in so many others of later date, it was from the botanical side that these epoch-making researches originated—a fact which may surprise many persons who have been accustomed to regard botany merely as a sort of systematic pulling to pieces of flowers.

Much later Treviranus, also a botanist, endeavoured to trace the most highly specialised cells with their frequently complicated shapes, back to a primitively simple form, and his work was extended, and his main results confirmed by the celebrated Von Mohl. But the zoologists and physiologists were on their side not inactive, though they came later into the field, and the importance of the cell as the unit of organic structure became clearly recognised in the case of animals as well as in that of plants. The names of Schwann, Johannes Müller, and Max Schultze will always remain famous in connection with the establishment of these important conclusions, whose effect was still more firmly to unite the animal and vegetable kingdoms as forming merely branches of one common organic whole.

The cell structure, so obvious in plants, and in many animal tissues, was, however, destined, from its very clearness, to lead to a temporary misapprehension as to the relative importance of the various parts. Moreover, the common comparison of the bodily structure, where the various cells are set apart for the performance of certain functions, to a house composed of rooms severally adapted for special purposes, appealed to the imagination and served to stereotype the error, more especially, as might have been expected, amongst the botanists. The cell framework absorbed the chief share of attention, to the protoplasm was hardly accorded even a second place. And yet it is precisely the protoplasm which is everything, and the cellular structure a later, and, so to speak, an accidental circumstance; useful, indeed, but not fundamentally essential. A study of the lower organisms had already revealed cases which were at variance with the commonly received opinions as to the necessary structure of living beings; but it is to Max Schultze that the credit is due of examining the whole matter from a critical standpoint, and arriving at clear and correct conclusions respecting it. He insisted, in a series of memoirs published about 1860, on the paramount importance of the cell-contents (protoplasm), as contrasted with the framework, and he marshalled a series of convincing examples, which clearly demonstrated the fact that not only could protoplasm exist without any special limiting membrane, but that in a number of organisms this was the habitual condition through life. He further adduces the facts, already noticed by older writers, that the protoplasm may escape bodily from its cell as a free swimming body, or "Swarm cell," as it is now often called.

Didymium Cencopus. Portion of the margin of a reticulate plasmodium after Cienkowski (magn. 100.)

But besides these facts, we know also of numerous plants, especially in certain (but by no means in all) groups of seaweeds in which the entire body consists, apparently, of one enormous cell. This may grow out at definite spots, and the various portions thus differentiated may closely simulate in external appearance the structure of a higher plant with its stem, leaves, and roots. And yet the whole seaweed consists still of an unchambered body, so that a small animal could traverse all the various organs without encountering any partitions to hinder its progress.

It may conduce to subsequent clearness if a description of some individual organisms be given which shall be severally illustrative of the three chief types which are represented amongst living beings, namely, the non-cellular, the unicellular, and the multicellular forms. And after dealing with these three body forms, we may proceed to inquire more closely into the actual characters of the protoplasm itself.

An admirable example of a "non-cellular," or naked, mass of protoplasm, which leads an independent existence, and which carries on in itself the ordinary vital functions of growth and multiplication, is to be found in the white blood-corpuscles which may be seen living and moving about amongst the red ones, when a drop of blood is examined under the microscope. But these white corpuscles are exceedingly small objects; a much more convenient and striking instance of living naked protoplasm is afforded by almost any species of that remarkable class of organisms known as Myxomycetes, one representative of which is not unfrequently to be found amongst the refuse of tan yards, where it is known in its fruiting condition as "Flowers of Tan." In its ordinary growing and actively living state it forms masses of a soft, slimy gelatinous consistency, and may be found creeping in irregular shapes in the damper layers of the tan heaps at some distance beneath the surface. Fig. 1 illustrates the general appearance presented by the living slimy mass (which is generally known in this condition as a *Plasmodium*), as it may be seen creeping about over damp bodies. It is easy, by keeping a piece of wet glass in contact with the edge of the plasmodium, to induce those portions near it to put out protuberances, and to spread over the glass surface, the latter may then be removed, together with the portion of the myxomycete which is upon it, and be transferred to the stage of the microscope where the movements may be studied in detail. But this preliminary step shows that protoplasm as it occurs in this organism is easily divisible, without prejudice to life; and under certain conditions this is true of the same substance even when occurring in cells. When watched under the microscope it is seen to consist of a clear external layer, and a more or less densely granular interior. Moreover, movements are constantly taking place within the mass, the internal granules betraying it by their constant change of position. Often they may be seen streaming to one place, and the clear peripheral layer bulges out at this spot, and thus a protuberance (or *pseudopodium*) is formed. The entire mass moves along by constantly streaming and pushing out larger or smaller pseudopodia in this way. As the protoplasm encounters nutritive substances in its path it envelopes them by the same process of streaming round them, and absorbs what may be of use, and finally moves away from the refuse.

If the protoplasm now be killed and treated with suitable reagents a number of nuclei become apparent, scattered through the mass. It is extremely probable, as will be seen later, that each of these nuclei forms a little centre of activity, which enables the protoplasm in some way to continue to carry on its normal functions. There is, in fact, a general relation between the nuclei and the whole mass, of such a nature that if a piece of protoplasm could be separated off, and contained no nucleus, it would cease to grow and would ultimately perish.

## Figures and Tables

**Figure f1:**